# Induction of ROS generation and NF-κB activation in MARC-145 cells by a novel porcine reproductive and respiratory syndrome virus in Southwest of China isolate

**DOI:** 10.1186/s12917-015-0480-z

**Published:** 2015-09-10

**Authors:** Yulin Yan, Aiguo Xin, Qian Liu, Hui Huang, Zhiyong Shao, Yating Zang, Ling Chen, Yongke Sun, Hong Gao

**Affiliations:** Faculty of Animal Science and Technology, Yunnan Agricultural University, Kunming, Yunnan People’s Republic of China; State Key Laboratory of Veterinary Etiological Biology, Lanzhou Veterinary Research Institute, Chinese Academy of Agricultural Sciences, Lanzhou, Gansu People’s Republic of China; Yunnan Animal Science and Veterinary Institute, Kunming, Yunnan People’s Republic of China

**Keywords:** PRRSV, ROS, NF-κB, MARC-145 cells

## Abstract

**Background:**

Porcine reproductive and respiratory syndrome virus (PRRSV) is the cause of an economically important swine disease that has devastated the swine industry since the late 1980s. The aim of the present study was to investigate the interaction between reactive oxygen species (ROS) and NF-κB by PRRSV infection.

**Results:**

We isolated the local strain of PRRSV from southwest China, designated YN-2011, then sequenced and analyzed the genome. YN-2011 was then used to evaluate the interaction of ROS and NF-κB. In PRRSV infected MARC-145 cells, there was a time-dependent increase in ROS and Maleic Dialdehyde (MDA). Accordingly, NF-κB activation was also increased as PRRSV infection progressed. Degradation of IκB mRNA was detected late in PRRSV infection, and overexpression of the dominant negative form of IκBα significantly suppressed NF-κB induced by PRRSV.

**Conclusions:**

The results indicate that the generation of ROS is involved in PRRSV replication and this progression is associated with the alteration in NF-κB activity induced by ROS. These results should extend our better understanding the interaction between PRRSV and host MARC-145 cells.

**Electronic supplementary material:**

The online version of this article (doi:10.1186/s12917-015-0480-z) contains supplementary material, which is available to authorized users.

## Background

Porcine reproductive and respiratory syndrome virus (PRRSV), the causative agent of porcine reproductive and respiratory syndrome in swine, is a positive-stranded RNA virus of the *Arteriviridae* family in the order *Nidovirales*. PRRSV causes highly significant losses to the swine industry worldwide as a result of both reproductive failure (late term abortions and stillbirths) in pregnant sows and respiratory disease (pneumonia) in pigs of all ages [1, 26, 38]. Infection with PRRSV also predisposes pigs to infection by bacterial pathogens as well as other viral pathogens, and is a key etiological agent of porcine respiratory disease complex [1, 13, 48]. PRRSV has a tropism for host cells of a phagocytic lineage, especially porcine alveolar macrophages (PAMs). The major obstacles to controlling PRRSV include genetic and antigenic variation of the virus and the dysregulation of the host response [10]. Despite extensive research, currently available PRRSV vaccines are not protective against all field strains, resulting in less-than-perfect control programs. Unraveling the mechanisms of PRRSV and cell interactions will be a critical step in developing predictable control and prevention strategies.

Viral infection is often accompanied by an alteration of the intracellular redox state. A feature of many viral infections includes increased generation of reactive oxygen species (ROS), which include superoxide, singlet O_2_, H_2_O_2_, and the highly reactive hydroxyl radical. ROS are often viewed as etiologic in producing cellular injury. Maleic Dialdehyde (MDA) is the peroxidation product of ROS and also a major marker of cell oxidative stress injury. ROS modulate the permissiveness of cells to viral replication and regulate host inflammatory and immune responses, ROS and MDA result in oxidative damage to both host tissue and the virus [15]. There are several reports documenting generation of ROS during viral infection. For example, infection with Herpes simplex virus, paramyxovirus, influenza virus, Hepatitis virus, or HIV, oxidative stress and free radical generation contribute to the pathogenesis of disease [30, 51]. ROS can attack many kinds of unsaturated fatty acids on biological membranes causing lipid peroxidation effects, which can induce cellular effects such as nuclear factor kappa B (NF-κB) activation [18, 20].

NF-κB belongs to a family of inducible transcription factors involved in pathogen- or cytokine-induced immune and inflammatory responses, as well as cell proliferation and survival [47]. The members of the NF-κB family in mammalian cells include p50/p105 (NFκB1), p65 (RelA), p52/p100 (NFκB2), c-Rel, and RelB. All of these proteins share a conserved 300-amino acid region known as the Rel homology domain that is responsible for DNA binding, dimerization, and nuclear translocation of NF-κB [25, 33]. Classical NF-κB exists as heterodimers consisting of a 50-kDa subunit (p50) and a 65-kDa subunit (p65) [22]. Under normal physiological conditions, NF-κB is sequestered in the cytoplasm as inactive complexes by its interaction with a member of the inhibitory kappa B (IκB) family. When stimulated with a wide range of pro-inflammatory stimuli, the IκB proteins are phosphorylated by IκB kinase (IKK) and degraded in proteasomes. The subunit of NF-κB p65 is then phosphorylated while exposing its nuclear localization signal sequence (NLS), leading to nuclear translocation and subsequent binding of NF-κB to DNA regulatory elements of the target genes involved in biological functions [3, 40]. The purpose of this study was to investigate the interaction between reactive oxygen species (ROS) and NF-κB during PRRSV infection in MARC-145 cells. The results of this study contribute to our understanding of the interaction between PRRSV and target cells from an oxidative stress point of view.

## Methods

### Ethics statement

This study was carried out in strict accordance with the recommendations in the Guide for the Care and Use of Laboratory Animals of the National Institutes of Health. The protocol was approved by the Committee on the Ethics of Animal Experiments of the Yunnan Agricultural University. All surgery was performed under sodium pentobarbital anesthesia, and all efforts were made to minimize suffering.

### Cells and virus

The MARC-145 cell line, a PRRSV permissive cell line, were cultured and maintained in Dulbecco’s Modified Eagle medium (DMEM) supplemented with 10 % heated-inactivated fetal bovine serum (FBS), 0.25μg/ml fungizone, 100U/ml penicillin, 10μg/ml streptomycin and 5μg/ml gentamicin and held at 37 °C in a humidified 5 % CO_2_ incubator.

The PRRSV isolate YN-2011 (GenBank accession no. JX 857698) was isolated and propagated in MARC-145 cells. The 50 % tissue culture infectious dose was determined using the Reed and Muench method. Virus stocks were prepared with a low passage isolate. For virus infection, cells were infected at a multiplicity of infection (MOI) of 1.

### Virus isolation

Tissue samples, including lung and lymph node (LN) were collected from pigs in the Yunnan province of Southwest China in 2011. Pigs displayed typical symptoms of PRRS, including labored breathing, pyrexia, lethargy, and anorexia. Standard methods for virus isolation were performed for lung and LN tissue homogenates. Briefly, serial 1:10 dilutions of homogenate were added to confluent MARC-145 cells. Cells were maintained at 37 °C with 5 % CO_2_ and monitored daily for cytopathic effects (CPE). The culture supernatants were harvested when CPE appeared in 70 % of the cells. The isolated PRRSV stock, termed YN-2011, was stored at −70 °C for further experiments.

In addition, an indirect immunofluorescence assay (IFA) was carried out with the commercial PRRSV monoclonal antibody SDOW-17 (RTI, USA). MARC-145 monolayer cells were washed once with PBS, fixed in 80 % acetone for 5 to 10 min, and air dried. For detection of the PRRSV, the primary antibody SDOW-17 and secondary antibody goat anti-mouse IgG-fluorescein isothiocyanate (FITC) conjugate (Sigma, USA) were added to each well respectively. After 30 to 60 min of incubation, the antibody was aspirated and monolayers were washed three times with PBS prior to viewing under a fluorescence microscope.

### Primer design and RT-PCR

In order to determine the full-length genomic sequence of the PRRSV isolate designated YN-2011, primers were first selected based on published sequences of the North American prototype VR-2332 (GenBank accession no. U87392). Viral RNA was extracted using TRIzoL Reagent (Invitrogen, CA, USA), dissolved in nuclease-free water, and stored at −70 °C until further use. Both the reverse transcription and the polymerase chain reaction were conducted using the PrimeScript One Step RT-PCR Kit (TaKaRa, Japan) according the manufacturer’s directions. Briefly, reverse transcription was performed at 50 °C for 30 min in the presence of a reaction mixture consisting of 2.5 μl of the RNA, 0.5 μl of forward and reverse primers, 1μl PrimeScript One Step Enzyme mix, 8 μl RNase-free H_2_O, and 12.5 μl 2× One Step Buffer. The cycling conditions were 94 °C for 2 min, followed by 40 cycles of denaturation (94 °C for 10 s), annealing (58 °C for 30 s), and extension (72 °C for 30 s), followed by a final extension at 72 °C for 7 min.

### Genome cloning, sequencing, and analysis

The amplified PCR products were analyzed by gel electrophoresis then purified using the Agarose Gel DNA Purification Kit (TaKaRa, Japan). The PCR products were cloned into the pMD19-T vector according to the manufacturer’s instructions (TaKaRa, Japan). Multiple clones of each PCR product were sequenced, and the consensus of each fragment were assembled to determine the complete genome of YN-2011.

The complete genomic sequence was compared with other isolates by phylogenetic tree, which was generated by the distance based Jotun Hein method using Lasergene v7.1 software (DNASTAR). The evolution of YN-2011 was analyzed by comparison with other known isolates worldwide. Multiple-sequence alignments were generated with MegAlign, and nucleotide sequence homologies were further assessed with Lasergene.

### Measurement of ROS and MDA

Intracellular ROS levels were measured with 2′,7′-dichloro-fluorescein- diacetate (DCFH-DA; Beyotime, China) as previously described. DCFH-DA passively diffuses into cells and is de-acetylated by esterases to form non-fluorescent 2′,7′-dichlorofluores-cein (DCFH). In the presence of ROS, DCFH forms the fluorescent product DCF, which is trapped inside the cells. MARC-145 cells were seeded at a density of 1 × 10^4^/well in 96-plate (Corning, USA) and cultured until the cells reached approximately 70-80 % confluence. Cells were then infected with PRRSV at a MOI of 1and incubated for 12 h, 24 h, 36 h, 48 h and 60 h, respectively. To obtain dissociated MARC-145 cells for the ROS assay, culture medium was first removed and the cells were washed three times with PBS. DCFH-DA, diluted to a final concentration of 10 μM with a serum free DMEM, was added to cultures and incubated for 30 min at 37 °C. The fluorescence was measured at 485 nm for excitation and 530 nm for emission with a fluorescence plate reader (TECAN infinite M200, TECAN). Measurement of the lipid peroxidation marker MDA was performed using a lipid peroxidation kit (Nanjing jiancheng, China), according to the manufacturer’s instructions.

### Western blot

Standard methods were used to perform Western blot. Briefly, cytoplasmic or nuclear extracts were diluted (1:2) in 2× sample buffer and boiled for 5 min. Twenty micrograms of each extract was subjected to sodium dodecyl sulfate-polyacrylamide gel electrophoresis (SDS-PAGE) and transferred to a nitrocellulose membrane (Amersham Biosciences, USA). The membrane was washed with phosphate-buffered saline-Tween 20 (TPBS), blocked in a solution of TPBS containing 5 % nonfat dry milk (blocking buffer), and then washed three times. The membrane was then incubated with primary antibody diluted 1:200 in blocking buffer overnight at 4 °C, washed three times with TPBS, and incubated with the secondary antibody conjugated with horseradish peroxidase (HRP) diluted 1:10,000 in blocking buffer for 1 h at RT. Samples were washed three times with TPBS, and then the signal was detected with the chemiluminescent protein detection system according to the manufacturer’s directions (Amersham Biosciences, USA). Antibodies used for Western blot include anti-NF-κB p65 (Santa Cruz Biotechnology, USA), anti-rabbit IgG-HRP (Amersham Biosciences, USA), and anti-actin (Sigma, USA).

### RNA extaction and quantitative real-time RT-PCR

Total cellular RNA was extracted from PRRSV-infected MARC-145 cells using an RNAprep pure cell kit (Qiagen, USA). RNA (0.4 μg) was reverse transcribed in a 20 μl reaction mixture. The cDNA product was amplified in a 25 μl reaction mixture containing SYBR Green Real-time PCR Master Mix (Toyobo Co. Ltd., Japan). IκBα gene specific primers for real-time RT-PCR were 5′-TCCACTTGGCGGTGATCA-3′ (forward), 5′-ATCACAGCCAGCTTCCAGAAG-3′ (reverse). β-actin gene specific primers were 5′-TGAGAACAGCTGCATCCACTT-3′ (forward), 5′-CGAAGGCAGCTCGGAGTT-3′ (reverse). Each cDNA sample was performed in triplicate. PCR amplifications were performed using a Roche Light Cycler 480 Real-Time System (Roche, Switzerland). Thermal cycling conditions were 10 min at 95 °C and 40 cycles of 10 s at 95 °C, 30 s at 58 °C and 15 s at 72 °C. In the present study, the data are presented as the change in target gene expression in stimulated MARC-145 cells that are normalized to the internal control gene (18S rRNA) and relative to the mock control expressed by the 2^-∆∆*CT*^ method [35]. Increased mRNA expression was defined as a change of ≥ 2.0-fold, “normal” expression was a change ranging from 0.5001- to 1.9999-fold, and decreased mRNA expression was a change of ≤ 0.5-fold.

### Statistical analysis

The Student’s *t* test was used for the statistical analyses. *P* values of less than 0.05 were considered statistically significant.

## Results

### Isolation and characterization of YN-2011

In 2006, a highly pathogenic strain of type 2-PRRSV (HP), causing high fever and severe morbidity and mortality in pigs of all ages, emerged in swine farms all over China [44]. Since after several years, the PRRSV YN-2011 was isolate from one of the Yunnan province pig farm which pig presenting the similar clinical signs of HP-PRRSV, such as rubefaction, blood spots, petechiae, erythematous blanching rashes, and pimples, frequently observed in ears, mouth, noses, back, and the inner thigh. Other common symptoms included high fever (40–42 °C), depression, anorexia, cough, asthma, lameness, shivering, disorder in the respiratory tract, and diarrhea. The pig farm never vaccination with PRRSV before that.

**Fig. 1:**
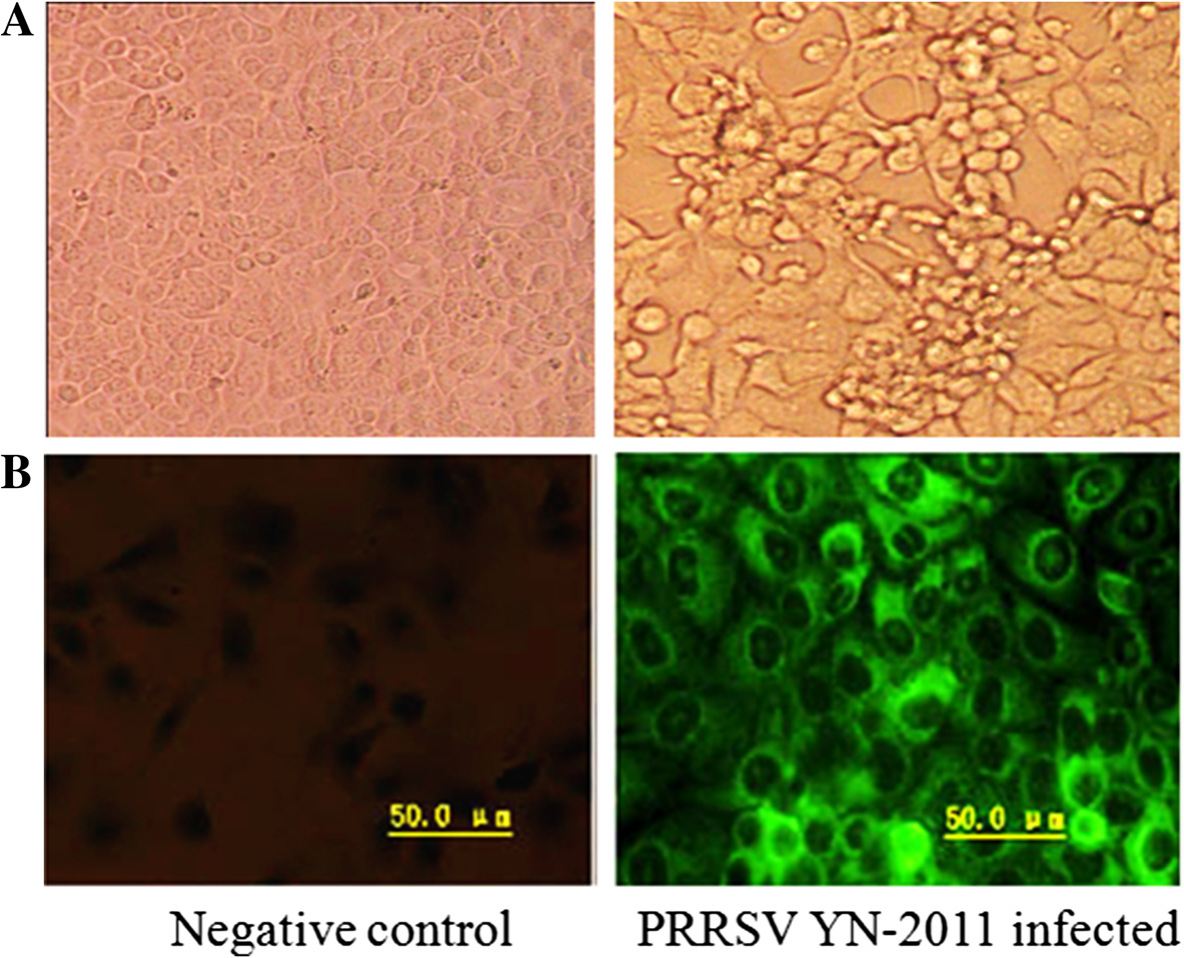
Negative control and PRRSV isolate YN-2011 infected MARC-145 cells. **a** PRRSV induced cytopathic effects (CPE) appeared approximately 72 h after infection. **b** Indirect fluorescent antibody staining with FITC, 48 h after infection, confirmed the presence of PRRSV. Pictures were taken with a Leica camera (Germany)

Approximately 72 h after exposure to lung or Lymphonode homogenates, MARC-145 cells presented with cytopathic effects (Fig. 1a). The presence of PRRSV was confirmed by staining with the monoclonal antibody SDOW-17 (Fig. 1b).

**Fig. 2:**
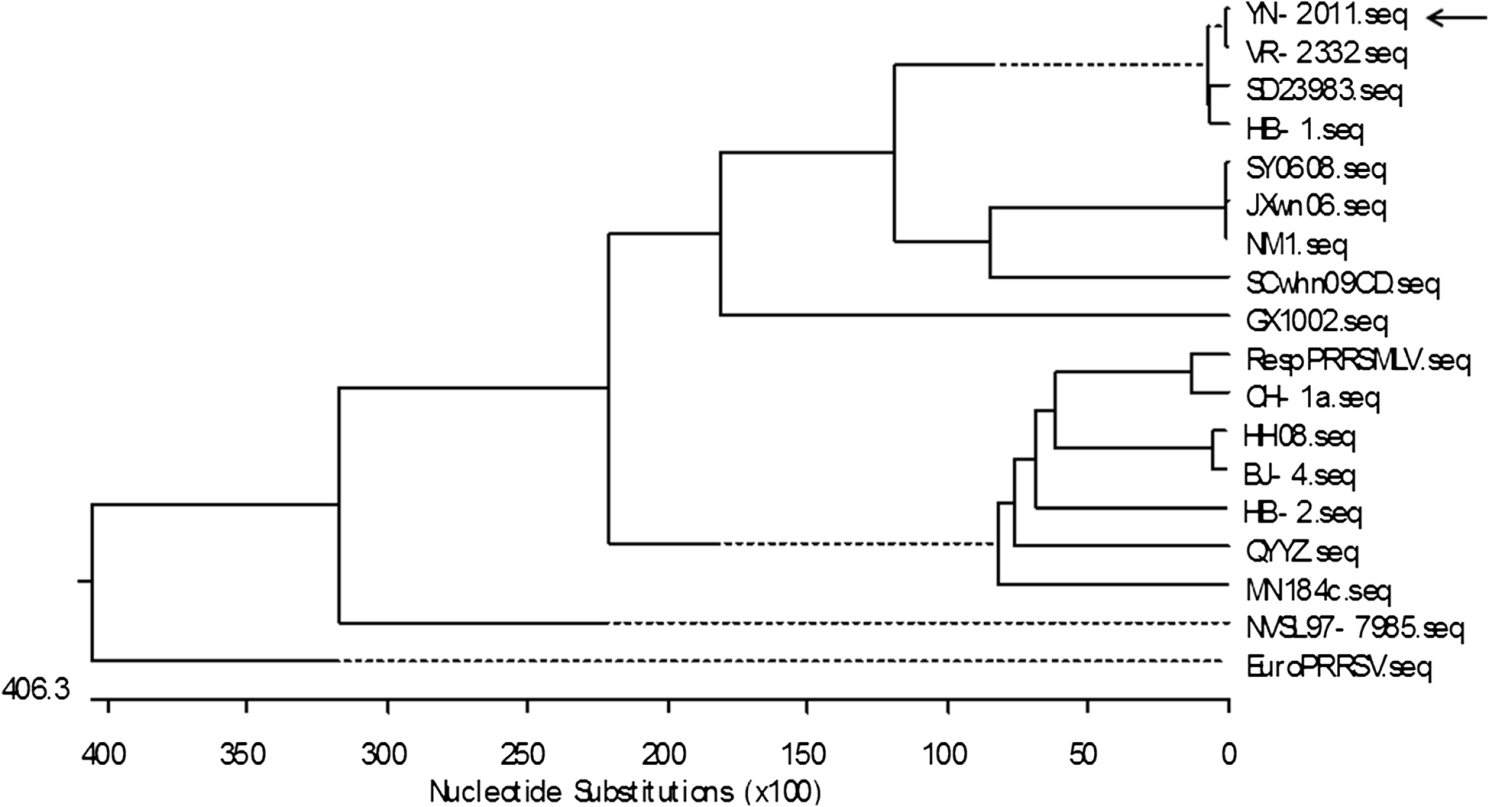
Phylogenetic analysis of YN-2011. The tree was constructed using the complete genomic sequences of YN-2011 and other PRRSV sequences downloaded from GenBank. Multiple-sequence alignments were performed using the DNASTAR. The tree was constructed by the Jotun Hein method using Lasergene. v 7.1 software. The scale bar shows nucleotide substitution (×100). The arrow indicates the location of YN-2011

YN-2011 was compared with the full length nucleotide sequences of other PRRSV isolates (Fig. 2) Additional files 1, 2 and 3. Phylogenetic analysis showed that PRRSV divided into two major genotypes, with the YN-2011 isolate belonging to type 2. Within the type 2 branch, several minor branches were observed. The results indicate YN-2011 most closely resembles VR-2332 (GenBank accession no. U87392), with a nucleotide identity of 99.5 %. Compared with previous Chinese isolates, YN-2011 shared only 89.2 % identity with HB 1(sh)/2002, 73.6 % identity with BJ-4, 71 % identity with CH-1a, 73.4 % identity with HB-2(sh)/2002, and 62.8 % identity with the highly pathogenic strain JXwn06 respectively. Furthermore, YN-2011 did not possess the 30-aa deletion within Nsp2 commonly associated with HP-PRRSV [45].

### PRRSV infection of MARC-145 cells induces ROS and MDA production

**Fig. 3:**
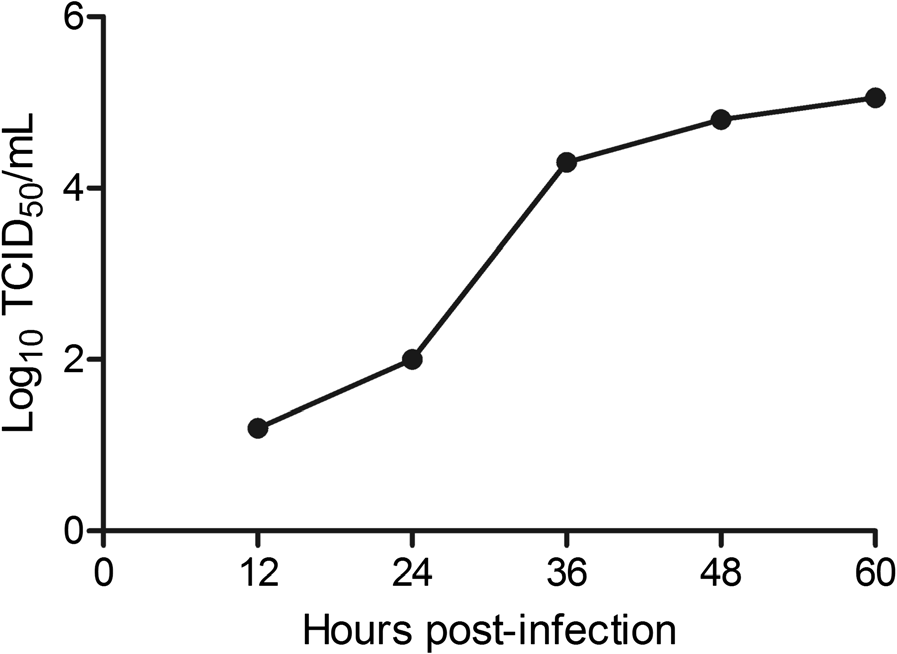
Growth kinetics of YN-2011 in MARC-145 cells. Cytopathic effects (CPE) were observed on MARC-145 cells after infection by viruses for 3 days. The cell supernatants were collected at 12, 24, 36, 48 and 60 hpi for virus titration in MARC-145 cells

**Fig. 4:**
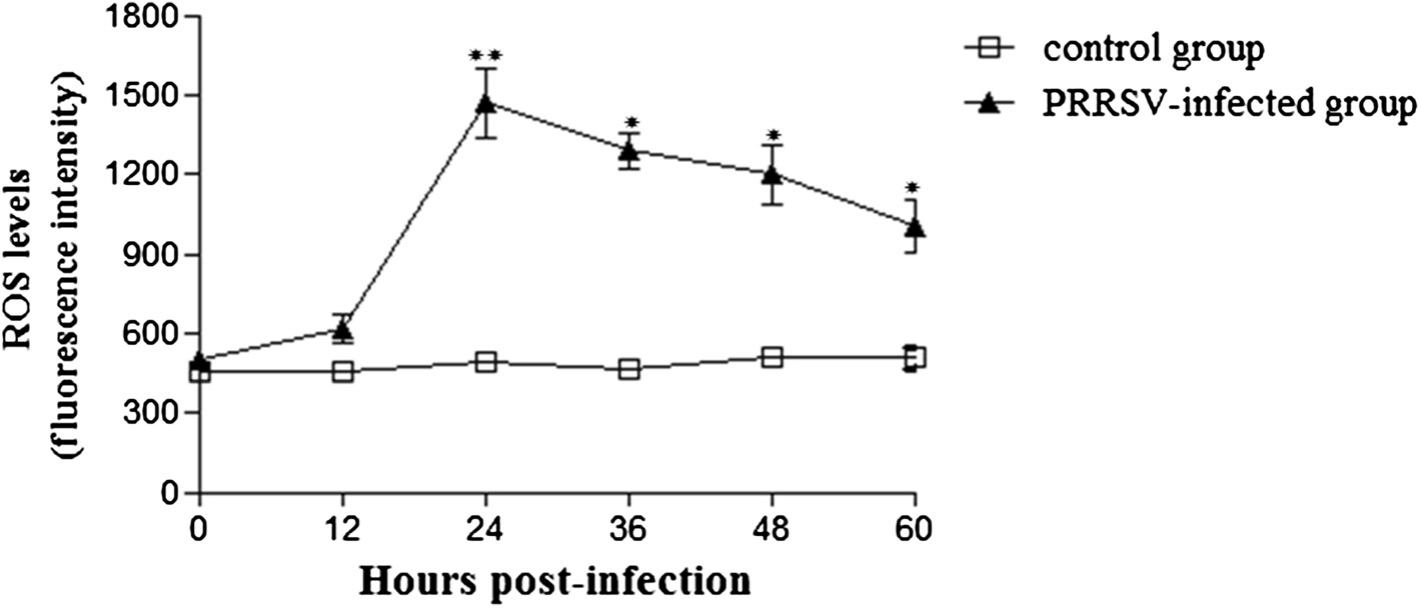
ROS production following PRRSV infection. Supernatants were collected at different time points after PRRSV infection in MARC-145 cells. At the time of collection, DCFH-DA was added to cultures and incubated for 30 min at 37 °C. Fluorescence intensity was measured within 60 min at 485 nm for excitation and 530 nm for emission. *P < 0.05, **P < 0.01

The growth kinetics of YN-2011 in MARC-145 cells was observed (Fig. 3). To determine whether PRRSV infection induced ROS production, MARC-145 cells were infected with PRRSV at MOI of 1, and supernatants were collected at different time points. DCFH-DA was added to cultures at each collection time point and incubated for 30 min at 37 °C. Fluorescence was measured within 60 min. ROS levels were similar for control and infected groups at 0 and 12 h time points (Fig. 4). Significantly higher levels of ROS were detected at 24, 36, 48, and 60 h time points in PRRSV infected cells compared to controls, with ROS levels peaking at 24 h post infection (hpi). As a positive control for DCF-DA oxidation, MARC-145 cells were exposed to 100 μM ROSUP (Beyotime, China) for 20 min. Similar to PRRSV infected cells, exposure to ROSUP resulted in a significant increase in mean fluorescent intensity compared to un-stimulated cells (data not shown). These results demonstrate that the production of ROS was increased following PRRSV infection.

**Fig. 5:**
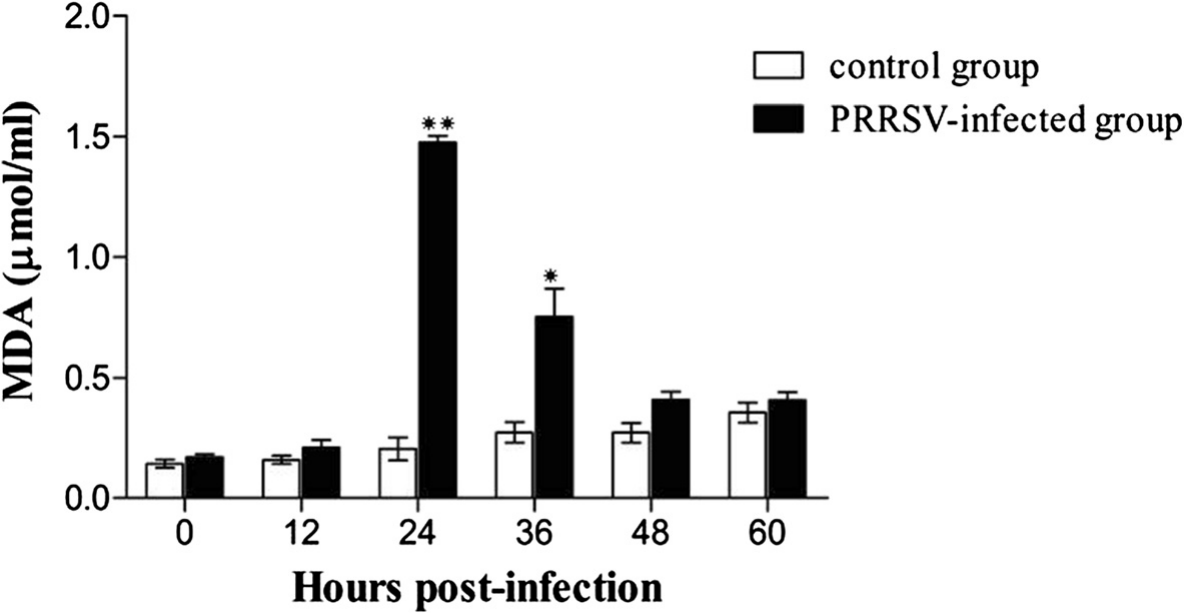
MDA production following PRRSV infection. Cell supernatants were collected at different time points after PRRSV infection in MARC-145 cells. MDA levels were measured using a commercial kit (Nanjing jiancheng, China) according to the manufacturer’s instructions. *P < 0.05, **P < 0.01

To confirm production of ROS levels, MDA concentrations were evaluated. Similar to the results of ROS production, there was a time-dependent increase in MDA production in PRRSV-infected MARC-145 cells compared with control cells (Fig. 5).

### NF- κB activation during PRRSV infection

**Fig. 6:**
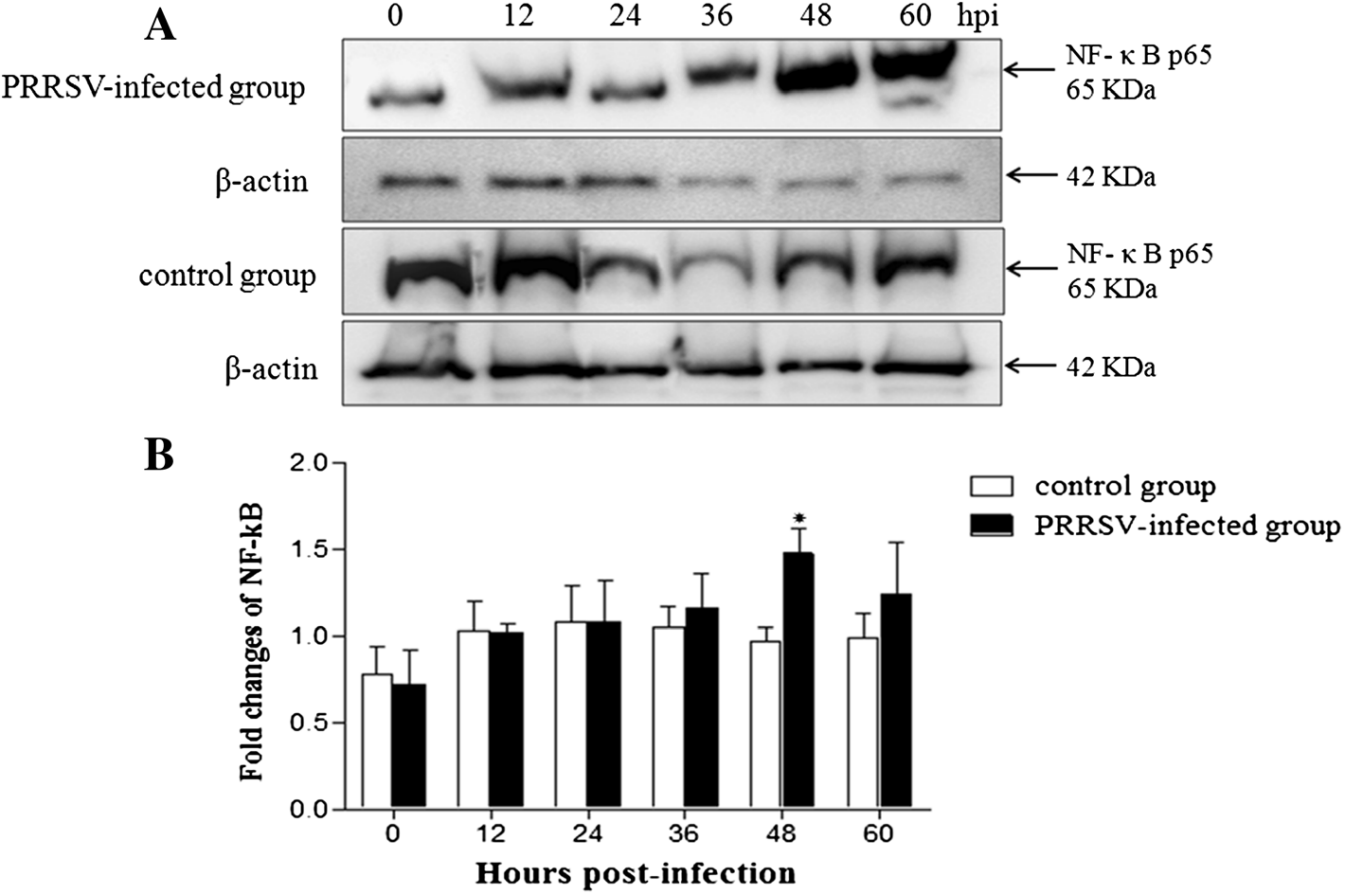
Expression of p65 protein following PRRSV infection in MARC-145 cells. MARC-145 cells cultures were infected with PRRSV at MOI = 1. Cytoplasmic extracts were prepared at indicated time points and subjected to Western blot analysis with antibodies specific for p65, a key protein for NF-κB activation. β-actin was included as a control for sample loading. **a** The p65 protein expression was examined by Western blot. **b** The band intensity was detected and the fold changes of NF-κB were calculated compared with control group. *P < 0.05

p65 protein expression, which is one of the key steps during activation of NF-κB, was detected by western blot following infection with PRRSV. As shown in Fig. 6, infection with PRRSV led to an increase in p65 protein in the nucleus, with significantly higher levels at 48 hpi compared to controls. In these (and subsequent) experiments, cell viability was assessed by trypan blue staining, and cultures were consistently found to be approximately 96 % viable in mock infected cells and 92 % in PRRSV-infected cells at 48 hpi (data not shown).

### PRRSV induced degradation of IκBα gene expression is dependent on infection time

**Fig. 7:**
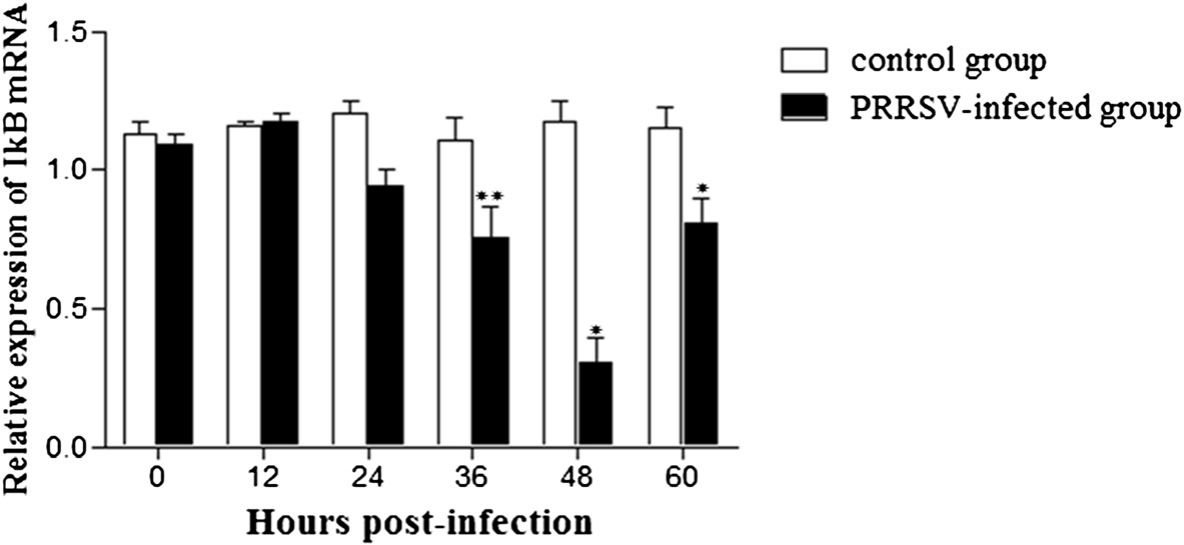
IκBα mRNA levels in MARC-145 cells infected with PRRSV. Cultures were infected with PRRSV at MOI = 1. Cells were collected at the indicated time points, and the endogenous transcription of IκBα mRNA was analyzed by real-time RT-PCR. The level of IκBα was first normalized to that of β-actin in the same sample and then compared with the control cells. *P < 0.05, **P < 0.01

Activation of NF-κB is characterized by degradation of IκBα after phosphorylation by IKK in response to many types of extracellular stimuli [39, 52]. This is followed by the phosphorylation of the NF-κB subunit p65 and nuclear translocation of NF-κB. To investigate the different time and potential mechanism(s) of NF-κB activation by PRRSV, IκBα gene expression levels were examined using quantitative real-time RT-PCR. As demonstrated in Fig. 7, IκBa was detected in both PRRSV infected and uninfected MARC-145 cells at 0, and 12, and 24 hpi. However, there was a significant decrease in IκBa mRNA levels at 36, 48, and 60 hpi in PRRSV infected MARC-145 cells compared to uninfected controls. The lowest level of IκBa mRNA was detected at 48 hpi, which correlated with the highest level of NF-κB activity.

## Discussion

PRRSV is characteristic of genetically extensive variation with the genetic/antigenic diverse strains in the field [24, 43, 56]. In the current study, we isolated and characterized YN-2011, which is the first PRRSV isolate identified in the Yunnan province of China. The complete genome was sequenced and submitted to the GenBank (accession no. JX 857698). Phylogenetic analysis indicated that the YN-2011 was most similar to the Type 2 isolate VR-2332, which shared 99.5 % identity at the nucleotide level and clustered together in a subgroup compared to other Type 2 and Chinese isolates. Interestingly, in contrast to the HP-PRRSV strain previously reported in China, the YN-2011 strain lacks the 30-aa deletion in Nsp2. However, YN-2011 could also lead to severe clinical signs in the field and possesses several new Nsp2 aa substitutions, such as 110-T/I, 284-S/F, 344,345-EV/QL, 474-P/S, 490-T/A, 565-Y/H, 567-D/N, 599-H/R, 683-R/H, 767-M/K, and 925-F/L [53]. The role of the new substitutions and virulence remains to be determined in the future. The results identify the viewpoint that the 30-amino-acid (aa) deletion in Nsp2 is not related to the virulence of the virus [57]. Our study clearly indicates that the HP-PRRSV variant, regardless of the 30-aa de-letion, continues to have a prevailing and accelerating evolution in China.

ROS and free radicals have been demonstrated to function as cellular signaling molecules, influencing a variety of molecular and biochemical processes [34]. These include expression of proinflammatory mediators, such as cytokines and chemokines. However, excessive ROS formation can lead to a condition of oxidative stress, which has been implicated in the pathogenesis of several acute and chronic respiratory diseases, such as asthma and chronic obstructive pulmonary disease (COPD) [5]. The induction of ROS has been observed following stimulation with a variety of molecules and infection with certain viruses like HIV, Hepatitis B, influenza, rhinovirus and respiratory syncytial virus [11, 14, 31]. In the present study, we investigated whether PRRSV infection in MARC-145 cells resulted in a condition of cellular oxidative stress, defined as a disruption of the pro-oxidant–antioxidant balance. Results showing a progressive increase in ROS (Fig. 4) and the lipid peroxidation product, MDA (Fig. 5), provide strong evidence of increased oxidative stress in PRRSV-infected MARC-145 cells. However, increasing ROS didn’t inhibit PRRSV replication in MARC-145 cells (Fig. 3), which indicated that infection and replication of PRRSV was independent with ROS. Previous reports have suggested that oxidative stress induced by increased generation of ROS is involved in the activation of NF-κB [12, 41]. Virus infections such as porcine circovirus type 2 (PCV2) [2], human immunodeficiency virus (HIV) [36], influenza virus [14, 29], hepatitis C virus (HCV) [23], Japanese encephalitis virus [46], and herpes simplex virus [7] activate the NF-κB pathway through ROS production. In this study, PRRSV infection indeed resulted in the generation of ROS firstly, and subsequent activation of NF-κB, which inducing the ROS level peak appeared ahead of 24 h with NF-κB activation peak (Figs. 4 and 6). The previously reported that the increased ROS production by H_2_O_2_ initiated NF-κB activation [19], but decreased the ROS could cause reduce the NF-κB expression [16].

Viruses have developed various strategies which lead to either activation or inhibition of NF-κB-dependent gene transcription [37]. The NF-κB pathway can be activated as a protective response of the host to viruses. Therefore, some viruses, such as vaccinia virus, African swine fever virus, influenza A virus, and mengovirus, have evolved strategies to block NF-κB activation in order to evade the innate immune response [42, 58]. The NF-κB pathway can also be activated directly by viruses. For example, HIV, herpesviruses, hepatitis C virus, encephalomyocarditis virus, reovirus, dengue virus, West Nile virus, and herpes simplex virus have evolved strategies to activate and exploit NF-κB for optimal replication, or to control host cell proliferation and survival to maximize viral progeny production [6, 8, 17, 49, 50]. Viruses modulate NF-κB activation through various mechanisms. Activation of NF-κB is usually mediated by degradation of IκBα in a proteasome-dependent mechanism after phosphorylation by IKK [9]. IκBα is generally thought to be the major inhibitor of NF-κB activation. NF-κB activation by influenza virus is mediated by oxidative radicals and activation of IKK as a result of over expression of viral proteins in endoplasmic reticulum [54]. The Tax transactivator oncoprotein of human T-lymphotropic virus-1 activates NF-κB by interacting directly with IKK [32]. HSV-1 induces persistent translocation of NF-κB by IκBα degradation [28].

Some viruses activate the NF-κB pathway through viral protein-cellular receptor interaction. In PPRSV infection in vitro, the nonstructural protein 2 (nsp2) and nucleocapsid protein (N) of PRRSV played the crucial role in NF-κB activation [4, 27, 43]. Previously, Lee and Kleiboeker showed that PRRSV activated the NF-κB pathway through IκBα degradation at 48 hpi [21]. NF-κB activation changed through IκBα degradation in vitro [4, 21]. In this study, similar results were observed, including activation of NF-κB at 48 hpi (Fig. 6). In addition, a decrease in IκBα expression levels was observed at 36 hpi (Fig. 7). Combined with the results for ROS and MDA production (Figs. 4 and 5), we suppose that ROS generation either directly or through activation of cellular signal pathway leads to down regulation of IkBα and the subsequent activation of NF-κB. A previous study has shown that blocking ROS by antioxidant N-acetyl-L-cysteine and tylvalosin abolished PRRSV-induced NF-κB activation but didn’t significantly affect PRRSV replication [21, 55]. Therefore, we postulate that at late stage, after the virus established its replication in host cells and maximized the viral progeny production, the virus would trigger the activation of NF-κB by ROS for inducing host cell apoptosis, which results in the releasing of mature viral particles. Future studies will confirm if PRRSV activates the NF-κB pathway in PAMs and in vivo. Understanding the role of NF-κB activation and its cascade signal pathway following PRRSV infection will contribute important information about the molecular pathogenesis of PRRSV infection.

## Conclusion

To our knowledge, this is the first time to report the PRRSV YN-2011 isolate from Yunnan province, southwest of China. The ROS generation involved NF-κB activation with time dependent in MARC-145 cells infected with YN-2011, which should extend our better understanding the interaction between PRRSV and host MARC-145 cells.

## Additional files

Additional file 1:
**Each ORF initial site of YN-2011.**
Additional file 2:
**The whole genome of YN-2011.**
Additional file 3:
**Each ORF gene nucleotide sequence of YN-2011.**

## Abbreviations

PRRS: Porcine Reproductive and Respiratory Syndrome
PRRSV: Porcine Reproductive and Respiratory Syndrome Virus
ROS: Reactive oxygen species
MDA: Maleic Dialdehyde
NF-κB: Nuclear factor kappa B
IκB: Inhibitory kappa B
